# Self-powered piezoelectric microfluidic flow sensor for low-flow monitoring of metal-ion solutions

**DOI:** 10.1039/d6ra02026g

**Published:** 2026-04-16

**Authors:** Yunzheng Zhang, Tao Wang, Jun Zheng, Wenjin Luo, Zhangjun Lan, Binyou Xie, Shushu Chen, Xinming Xia, Liuhua Mu, Jie Jiang, Yan Fan, Liang Chen

**Affiliations:** a College of Optical, Mechanical and Electrical Engineering, Zhejiang A&F University Hangzhou 311300 China fanyan503@zafu.edu.cn; b School of Physical Science and Technology, Ningbo University Ningbo 315211 China jiangjie1@nbu.edu.cn; c College of Physics Science and Technology, Yangzhou University Jiangsu 225009 China; d Noncommissioned Officer Academy of PAP Hangzhou 311400 China

## Abstract

Microfluidic technology enables precise manipulation of fluids at the microscale, where accurate flow velocity measurement is crucial for controlling mass transport, ion migration, and electrochemical responses. However, existing pressure sensors mainly respond to high-frequency dynamics or require external excitation, which limits stable detection under low-frequency or low-flow conditions. Here, we present a self-powered piezoelectric microfluidic flow sensor that detects flow rates as low as ∼3 µL min^−1^ over a broad measurable range of 3–203 µL min^−1^. Using a commercial piezoelectric film coupled with a PDMS membrane, the device converts diaphragm deformation into voltage signals without external power, achieving a high sensitivity 0.79 mV (µL min^−1^)^−1^, rapid response (0.1) ms, and excellent stability. The proposed sensor also offers low cost and scalable integration, showing strong potential for portable lab-on-a-chip applications.

## Abstract 2

A self-powered piezoelectric microfluidic flow sensor enables stable monitoring of metal-ion solutions at ultralow flow rates. With a PDMS isolation membrane and piezoelectric film, it delivers sensitive, rapid, and power-free detection.

## Graphical abstract

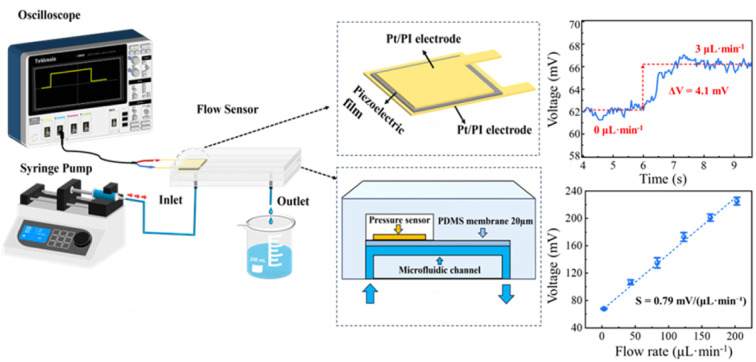

## 1. Introduction

Microfluidic technology has become a key technology in biomedical diagnostics,^1–3^ chemical analysis,^4,5^ and environmental monitoring, owing to its capability for precise manipulation of fluids at the microscale.^6–8^ Within such microfluidic systems, fluid transport characteristics are of fundamental importance for signal transduction. Notably, in systems involving metal-ion solutions, the flow velocity critically governs mass transport processes, metal-ion migration, and associated electrochemical responses.^9–13^ Consequently, accurate measurement of the flow velocity of metal-ion solutions is essential for reliable characterization and optimization of microscale systems.^14,15^ To address this need, there is a pressing demand for high-performance microfluidic sensors that simultaneously offer high sensitivity, rapid response, and excellent integrability, thereby enabling precise measurement of extremely small liquid volumes.^12,16,17^

Compared with other working fluids, metal-ion (electrolyte) solutions possess high ionic conductivity, which makes the electrical readout of microfluidic pressure/flow sensors more vulnerable to perturbations and long-term instability.^9,18,19^ Conductive electrolytes can promote parasitic electrochemical processes and electrode polarization, and when insulation is imperfect establish ionic leakage paths.^9,20^ This parasitic coupling typically manifests as pronounced baseline drift and elevated low-frequency (1/*f*) noise, severely degrading the signal-to-noise ratio.^21,22^ Meanwhile, prolonged operation leads to surface contamination and adsorption, which degrade signal stability and measurement repeatability.^23,24^ Consequently, even when sensors are mechanically compliant enough to respond to minute pressure variations, the weak pressure signatures associated with low-velocity electrolyte microflows are frequently obscured by drift and noise, rendering reliable sensing under long-duration, low-frequency, or low-velocity conditions particularly challenging.^12,15^ Therefore, microfluidic sensing for metal-ion solutions demands robust fluidic electrical isolation, suppressed thermal drift, and sustained operational stability. Nevertheless, despite extensive progress in optical, thermal, and electrical strategies, real-time and stable detection of low-velocity, low-frequency electrolyte microflows remains difficult.^12,25,26^

Among existing approaches, diaphragm-based sensors are attractive due to their simple architecture and high mechanical compliance, converting flow-induced pressure into electrical signals *via* piezoresistive, capacitive, or piezoelectric readouts.^25,27–29^ However, piezoresistive and capacitive schemes typically require external excitation and complex signal conditioning, which can be particularly undesirable in conductive electrolytes due to susceptibility to drift and interference.^9,18^ Meanwhile, triboelectric and many piezoelectric mechanisms respond predominantly to dynamic, high-frequency pressure variations, limiting their effectiveness for low-frequency or low-velocity microflows that are common in metal-ion microfluidic operation.^30,31^ A practical trade-off is thus often encountered between low-flow detectability and dynamic range.^12,26^

Here, we present a self-powered piezoelectric microfluidic flow sensor designed for stable monitoring of metal-ion solution microflows. The device integrates a commercial piezoelectric film with a thin PDMS isolation diaphragm that physically separates the working electrolyte from the sensing element while enabling efficient mechanical coupling. Flow-induced pressure deforms the diaphragm and generates an open-circuit voltage without external power or dynamic excitation, enabling reliable detection down to ∼3 µL min^−1^ over a broad range of 3–203 µL min^−1^. This electrically isolated, low-cost architecture facilitates scalable integration for portable lab-on-a-chip applications involving conductive metal-ion solutions.

## 2. Experimental section

### 2.1. Device configuration

**Fig. 1:**
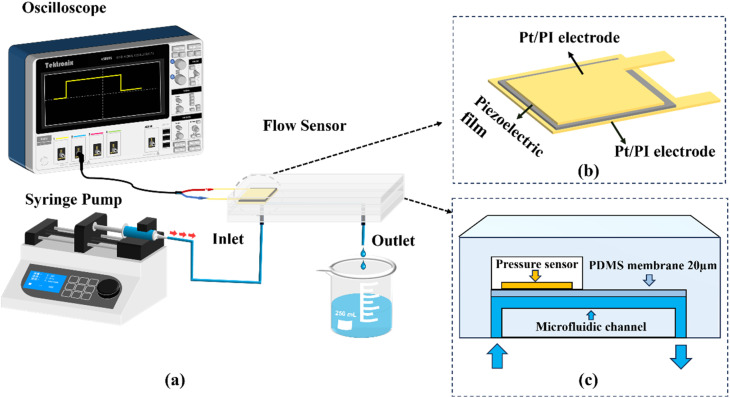
Design and configuration of the piezoelectric microflow sensing system. (a) Schematic of the experimental setup including a syringe pump, microfluidic chip, and oscilloscope for open-circuit voltage acquisition. (b) Structure of the sensing element assembled from a piezoelectric film and Pt/PI electrodes (bottom: 5 × 5 mm^2^; top: 3 × 3 mm^2^). (c) Cross-sectional schematic of the integrated device showing the microchannel, a 20 µm PDMS isolation membrane, and the sensing cavity beneath the sensing element.

The microfluidic sensing system (Fig. 1a) was assembled using a custom-fabricated PDMS microchip connected to a syringe pump (Ditron-tech, LSP01-2A) through a flexible catheter (inner diameter: 0.51 mm). A piezoelectric sensor (Fig. 1b), assembled by sandwiching a commercial piezoelectric film (ZJ, ZJGOPZ02) between two platinum electrodes, was integrated above the sensing region of the chip, where the bottom electrode measured 5 × 5 mm^2^ and the top electrode measured 3 × 3 mm^2^. During operation, flow-induced pressure in the microchannel deformed the PDMS diaphragm and transferred mechanical loading to the piezoelectric element, thereby generating electrical signals. The open-circuit voltage output was recorded using an oscilloscope (Tektronix MSO44, 4-BW-200).

### 2.2. Fabrication of the piezoelectric sensing element

The sensing element was assembled from a commercial piezoelectric film (ZJ, ZJGOPZ02) cut to the desired size. Platinum (Pt) electrodes were fabricated by magnetron sputtering onto custom 200 µm-thick polyimide (PI) substrates used for electrode deposition. The sputtering process was carried out under vacuum using a CIS400 sputtering instrument with a Pt target at a working distance of 25 mm. Each sputtering cycle lasted for 60 s, and the deposition was repeated five times, giving a total sputtering time of 5 min and resulting in an approximate Pt film thickness of 5 nm. A small current fluctuation of about 1–2 mA was observed during the magnetron sputtering process, which is considered normal for this deposition procedure. These conditions were selected to provide sufficient electrical conductivity while minimizing additional mechanical stiffness, thereby improving strain transfer from the isolation diaphragm to the piezoelectric film.

A bottom Pt/PI electrode (5 × 5 mm^2^) and a top Pt/PI electrode (3 × 3 mm^2^) were prepared, each incorporating a lead section for electrical connection. The piezoelectric film was then sandwiched between the two Pt/PI electrodes to form the piezoelectric sensing element. The assembled element was integrated with the microfluidic chip by positioning it above the sensing cavity and mechanically coupling it through a 20 µm-thick polydimethylsiloxane (PDMS) isolation diaphragm, which physically separated the working fluid from the piezoelectric layer while allowing diaphragm deformation to transmit flow-induced hydraulic loading for self-generated voltage output. Prior to experiments, each assembled device was electrically inspected to ensure reliable contact and to exclude short- or open-circuit failures. As shown in Fig. 1b, the electrode-film stack forms a compact and well-aligned sensing structure. The asymmetric electrode layout (bottom: 5 × 5 mm^2^; top: 3 × 3 mm^2^) intentionally leaves a lateral alignment margin, which helps prevent electrode overlap or shifting and reduces the risk of short-circuiting under compression. After assembly, the electrode-film stack was encapsulated with a 0.6 mm-thick polyimide (PI) tape to minimize humidity effects and facilitate handling during experiments.

### 2.3. Assembly of the integrated microflow sensor

A three-layer PDMS microchip (overall size: 3 × 2 × 0.5 cm^3^) was fabricated, as illustrated in Fig. 1c. Polydimethylsiloxane (PDMS; Sylgard 184, Dow Corning) was prepared by mixing the base and curing agent at a 10 : 1 weight ratio and cured in an oven at 85 °C. The bottom layer contained the microfluidic channels (width × height: 200 µm × 30 µm) patterned by soft lithography. The middle layer consisted of a commercial PDMS membrane (KYQ series, 20 µm thickness), which was bonded onto the channel layer by oxygen plasma treatment to serve as a fluid isolation barrier. For the top layer, the piezoelectric sensing element was aligned and bonded onto the PDMS membrane, and the assembly was finally sealed with a PDMS cover sheet featuring a cavity that matched the sensor footprint. This cavity provided sufficient clearance to enable free diaphragm deformation during operation while maintaining a leak-tight microfluidic interface.

### 2.4. Electrical and mechanical characterization

The mechanical loading and electrical output of the piezoelectric sensing element were characterized using a Mark-10 test system, consisting of a digital force gauge (Mark-10, FS05, 50 N capacity), a motorized test stand (Mark-10, F505H), and an oscilloscope (Tektronix MSO44, 4-BW-200). The piezoelectric sensor was mounted on a rigid sample stage and loaded in the normal direction using a circular compression platen (diameter: 0.8 cm) aligned with the active area to ensure consistent contact conditions. The motorized test stand provided displacement-controlled loading, while the force gauge served to record the applied force. Loading setpoints and profiles were controlled *via* IntelliMESUR software.

To capture the intrinsic transient response, beyond the mechanical bandwidth of the Mark-10 test stand, a custom dynamic loading platform was developed, which incorporated a programmable digital timing controller module with MOSFET power output. The module was powered by a DC supply (5 V) and generated repeatable on/off drive pulses to actuate a compact loading actuator (electromagnet) connected to a pressure head matched to the sensor's active area. This actuator delivered a pressure pulse of approximately 100 kPa to the sensing area. The open-circuit voltage output was recorded by the Tektronix MSO44 oscilloscope at a sampling rate of 1 MHz.

### 2.5. Flow-rate calibration

To verify the accuracy of the syringe pump over the investigated flow-rate range, gravimetric calibration was carried out at 3, 10, 50, 100, and 200 µL min^−1^. For each programmed flow rate, the dispensed liquid was collected over a fixed time interval and weighed using an analytical balance (Sartorius BSA224S-CW, max capacity 220.0 g, readability 0.1 mg). The actual flow rate was then calculated from the measured mass and collection time. Each condition was repeated five times.

**Fig. 2:**
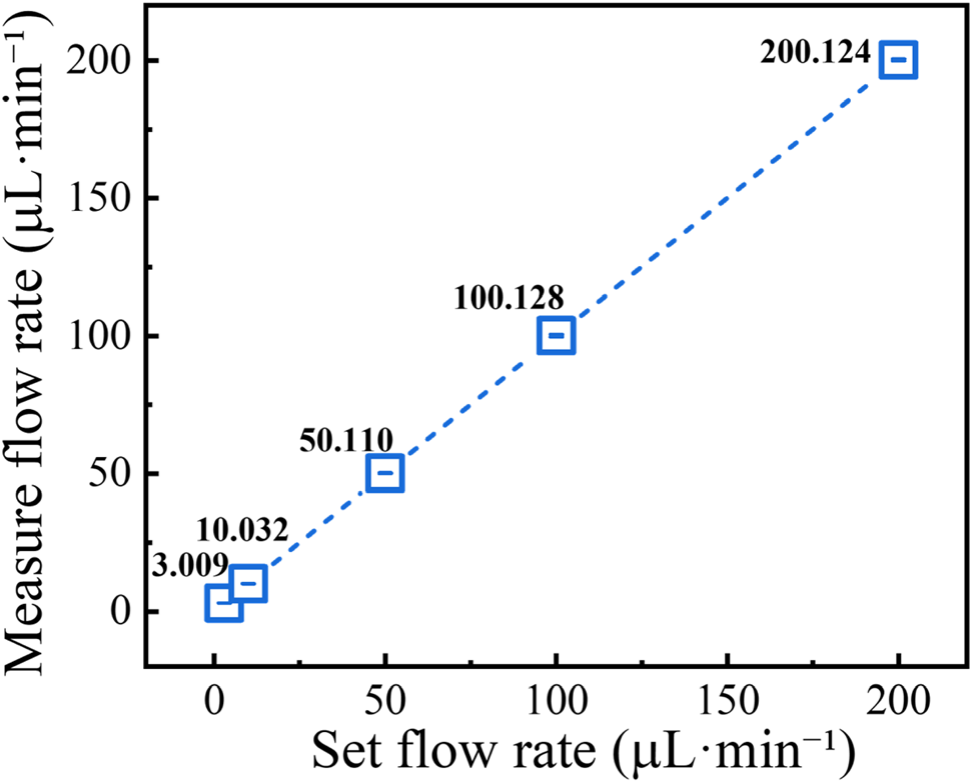
Gravimetric calibration of the syringe pump. Measured flow rates at 3, 10, 50, 100, and 200 µL min^−1^ showing linear agreement with the corresponding set values.

As shown in Fig. 2, the measured flow rates exhibited excellent linear agreement with the set values over the full calibration range. The detailed calibration results are summarized in Table S1 (SI). The measured flow rates were 3.009 ± 0.021, 10.032 ± 0.031, 50.050 ± 0.274, 100.088 ± 0.534, and 200.124 ± 0.192 µL min^−1^ (mean ± SD, *n* = 5), corresponding to mean relative errors of 0.30%, 0.32%, 0.10%, 0.09%, and 0.06%, respectively. In addition, the relative standard deviation remained low over the entire tested range, indicating good repeatability of the flow-delivery system.

These results confirm that the syringe pump provides reliable and accurate flow control under the operating conditions used in this work. Unless otherwise specified, a 1 mM NaCl solution was used as the working fluid in the subsequent microflow sensing experiments.

## 3. Results and discussions

### 3.1. Piezoelectric performance and stability

**Fig. 3:**
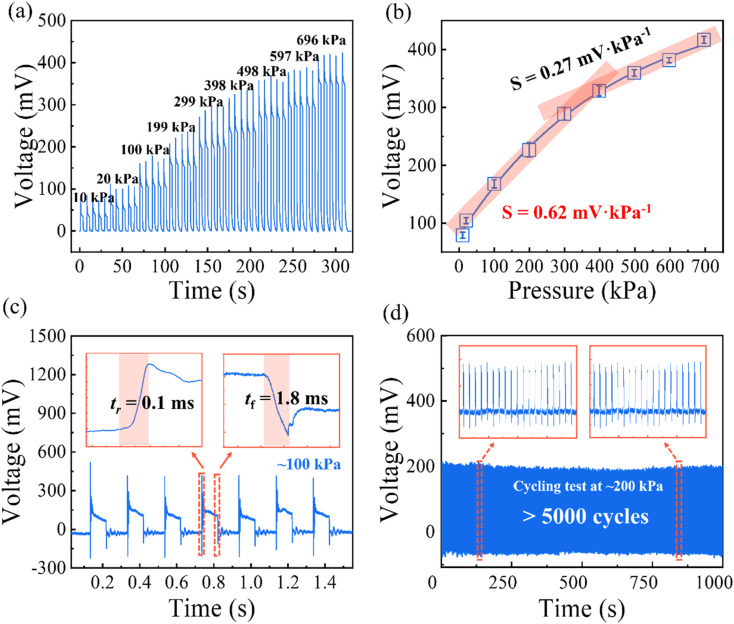
Electromechanical characterization of the piezoelectric sensing element. (a) Open-circuit voltage responses under stepwise normal loading (0.5–35 N) applied using a circular compression platen (diameter: 0.8 cm). The corresponding nominal contact pressures (10–696 kPa) were estimated from the measured force divided by the platen contact area. (b) Pressure-induced voltage output and corresponding sensitivity of the sensor at various pressure levels. (c) Transient response measured using a custom dynamic loading platform (pressure pulse: ∼100 kPa) to extract rise and recovery times. (d) Cyclic durability test at 200 kPa for 1000 s (5000 cycles). Voltage signals were acquired using an oscilloscope.

Before conducting the flow rate tests, we first performed preliminary electrical property measurements on the commercial piezoelectric film to better understand its electrical characteristics and response properties, which would provide insights for the subsequent flow sensing tests. The electromechanical performance of the piezoelectric element was first evaluated to quantify its pressure sensitivity, temporal response, and operational stability. The mechanical loading and electrical readout system consisted of a motorized test stand (Mark-10, F505H) equipped with a digital force gauge (Mark-10, FS05, 50 N capacity) and an oscilloscope (Tektronix MSO44 4-BW-200) for recording open-circuit voltage signals. The sensing element was fixed on a rigid stage and loaded in the normal direction using a circular compression platen (diameter: 0.8 cm) aligned to the active area. Stepwise normal forces of 0.5, 1, 5, 10, 15, 20, 25, 30 and 35 N were applied. For convenience, the corresponding nominal contact pressures were estimated from the platen area as 10, 20, 100, 199, 299, 398, 498, 597 kPa, and 696 kPa, respectively. As shown in Fig. 3a, the voltage output exhibited clear step-like transitions under both single-step and repeated loading, indicating stable and reproducible electromechanical transduction over the tested force range.

The device exhibited pronounced pressure sensitivity across the entire testing range. In the 10–398 kPa region, the output voltage increased stepwise from approximately 34 mV to 184 mV, corresponding to a sensitivity of 0.62 mV kPa^−1^. At higher pressures (398–696 kPa), the response remained linear but with a reduced slope of 0.27 mV kPa^−1^ (Fig. 3b). This indicates that, under direct mechanical loading, the incremental voltage response of the sensing element decreases at higher pressure, suggesting that the device is approaching its upper measurable loading range rather than exhibiting simple saturation. Such behavior reflects the reduced incremental electromechanical response of the sensing element under high loading conditions. We found that the response time obtained from low-frequency loading is limited by the mechanical loading rate of the Mark-10 test stand (2.5 N s^−1^), which prevents accurate extraction of the intrinsic rise and recovery times of the piezoelectric element. To capture the actual transient behavior, the sensing element was therefore tested using a custom dynamic loading platform controlled by a microcontroller (details provided in Section 2.4). A pressure pulse of approximately 100 kPa was applied using a pressure head matched to the sensing area, and the voltage output was recorded at a 1 MHz sampling rate using the Tektronix MSO44 oscilloscope. As shown in Fig. 3c, the transient response exhibited a rise time of 0.1 ms and a recovery time of 1.8 ms, demonstrating a fast electromechanical response of the piezoelectric element under the present test conditions.

To further evaluate the device performance beyond the transient response characterization, the repeatability and durability were assessed under cyclic loading. The sensing element was repeatedly loaded at a constant normal force of 200 kPa for 1000 s (Fig. 3d). The voltage output remained stable throughout the test and returned to its baseline level after each unloading event, indicating minimal drift. In addition, after 5000 loading cycles, no significant degradation in the signal amplitude was observed, confirming the mechanical robustness and long-term reliability of the piezoelectric film-based sensing element.

Additional material information was provided for the commercial piezoelectric film used in this work. The film had a thickness of approximately 100 µm and was supplied in a pre-poled state by the manufacturer. Since it was a commercial product, detailed crystallographic orientation information was not fully available from the supplier. To provide supplementary structural information, SEM characterization was performed. As shown in Fig. S1 (SI), the plan-view SEM image indicates a relatively smooth and uniform surface morphology, while the cross-sectional SEM image confirms a film thickness of approximately 100 µm.

**Fig. 4:**
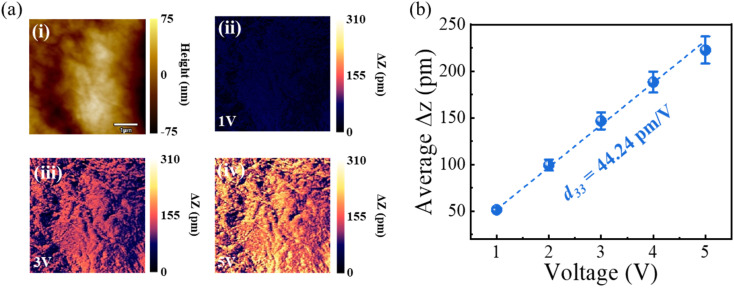
Piezoelectric Force Microscopy (PFM) characterization of the piezoelectric film. (a) Representative resonance response (amplitude) under different voltages. (b) Out-of-plane displacement (Δ*z*) as a function of AC voltage; error bars indicate the standard deviation from three different regions on the sample.

The commercial piezoelectric film exhibited excellent piezoelectric properties. To further investigate its piezoelectric nature, piezoelectricity measurements were conducted using Piezoelectric Force Microscopy (PFM) in Dual Amplitude Resonance Tracking (DART) mode with conductive silicon cantilevers (Olympus-AC240TM; tip radius: 28 nm; typical spring constant: 2 N m^−1^). Scanning was performed over an area of 5 µm × 5 µm. An AC excitation voltage ranging from 1.0 to 5.0 V was applied through the conductive tip, which induced measurable surface deformation of the piezoelectric film (Fig. 4a). The corresponding out-of-plane displacement (Δ*z*) increased linearly with the applied voltage (Fig. 4b). The calculated piezoelectric coefficient *d*33 was 44.2 pm V^−1^, indicating strong piezoelectric coupling and confirming the excellent electromechanical performance of the sensor film.

### 3.2. Microflow sensing and flow rate characterization

**Fig. 5:**
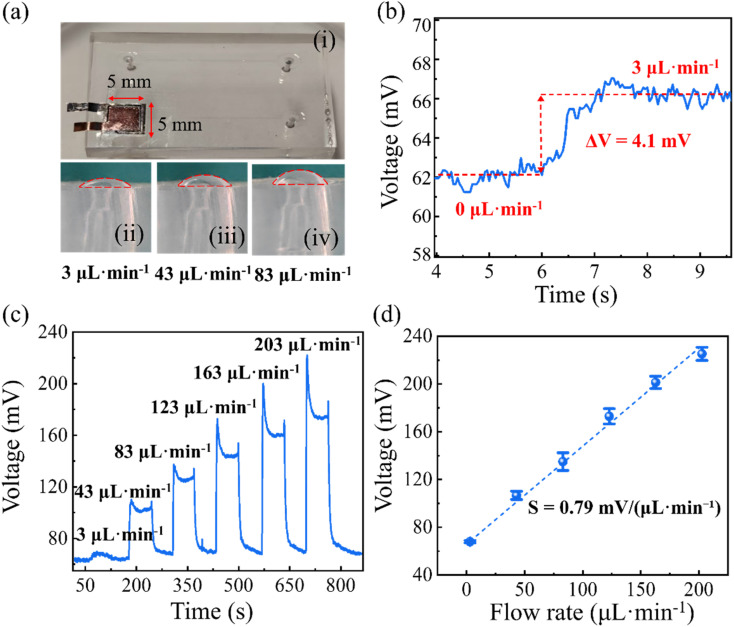
Flow-sensing performance of the integrated microfluidic sensor. (a) Photographs showing (i) the assembled device and (ii–iv) diaphragm deformation profiles at *Q* = 3, 43, and 83 µL min^−1^. (b) Representative voltage response at *Q* = 3 µL min^−1^ (Δ*V* = 4.1 mV). (c) Step-response voltage traces under pump-programmed flow-rate changes, demonstrating stable plateau outputs over *Q* = 3–203 µL min^−1^. (d) Linear correlation between voltage output and flow rate.

After the preliminary characterization, the piezoelectric element was integrated into a microfluidic chip to assess its flow-sensing performance. First, a 20 µm-thick PDMS isolation film was bonded to the microfluidic chip to prevent direct liquid contact, thereby avoiding potential device degradation and contamination. The piezoelectric sensor (5 mm × 5 mm) was then positioned at the inlet region of the microchip and affixed using polyimide (PI) tape. Additional PI tape was applied to ensure proper alignment and mechanical stability, completing the sensor-chip integration. This configuration enables effective mechanical coupling while maintaining full electrical and fluidic isolation of the sensing unit Fig. 5a(i).

To visualize diaphragm deformation more clearly under different flow conditions, the influence of flow rate on PDMS diaphragm deformation was examined at inlet flow rates of 3, 43, and 83 µL min^−1^. These conditions were carefully selected to provide observable deformation profiles while avoiding diaphragm rupture and maintaining partial cavity encapsulation for efficient pressure transfer. As shown in Fig. 5a(ii–iv), the PDMS membrane exhibited distinct deformation patterns at the selected flow rates. The pressure difference between the inlet region and the microchannel led to localized fluid accumulation near the inlet, causing elastic deformation of the PDMS film. With increasing flow rate, the induced pressure differential and consequently the strain experienced by both the diaphragm and the piezoelectric element increased accordingly. During flow measurements, the volumetric flow rate was directly controlled by the syringe pump, corresponding to the pump-programmed values, which were verified by gravimetric calibration over the investigated flow-rate range. As shown in Fig. 5b, the sensor produced a measurable voltage response (Δ*V* = 4.1 mV) at a low flow rate of 3 µL min^−1^. To further evaluate the detectability of the signal at the lowest tested flow rate, the signal-to-noise ratio (SNR) was calculated as SNR = Δ*V*/*σ*_*n*_, where Δ*V* is the signal amplitude and *σ*_*n*_ is the standard deviation of the baseline noise at 0 µL min^−1^. The calculated SNR was 15.3, corresponding to 23.7 dB, confirming that the output signal at 3 µL min^−1^ can be clearly distinguished from the background noise. Therefore, the minimum detectable flow rate was determined to be approximately 3 µL min^−1^ under the present measurement conditions. This result demonstrates that the microfluidic flow sensor configuration enables reliable detection of low-velocity electrolyte microflows without external power.

Step changes in flow rate were generated by directly adjusting the syringe pump setpoint across a series of flow-rate levels. Each transition typically produced an initial transient voltage peak, which is attributed to the rapid pressure redistribution and diaphragm deformation during pump ramping. After the flow stabilized at the target setpoint, the voltage evolved into a steady plateau value. As shown in Fig. 5c, the output exhibited clear step-like changes and stable plateau levels over the investigated flow-rate range of 3–203 µL min^−1^, indicating repeatable and robust sensing performance under pump-controlled operation.

The linear relationship between voltage output and flow rate is shown in Fig. 5d. Multiple repeated measurements were performed, and the standard error was analyzed. The flow-rate sensitivity *S*_Q_ of the device was defined as the change in voltage per unit change in flow rate:

1 *S* = Δ*V*/Δ*Q*

where Δ*V* represents the voltage variation induced by the change in flow rate, and Δ*Q* is the corresponding change in flow rate. The sensor exhibited a high flow-rate sensitivity of 0.79 mV (µL min^−1^)^−1^, demonstrating its capability to detect minute flow variations with high precision.

To further clarify the sensing mechanism, we replaced the previous semi-empirical elimination of two fitted sensitivities with a first-order physically grounded interpretation. For a steady pressure-driven Newtonian liquid in a rectangular microchannel operating in the low-Reynolds-number regime, the channel pressure drop follows a Poiseuille-type relation:^32,33^

2 Δ*P*_ch_ = *R*_h_*Q*

where Δ*P*_ch_ is the channel pressure drop, *R*_h_ is the hydraulic resistance, and *Q* is the volumetric flow rate. For a fixed channel geometry and liquid viscosity, the hydraulic load generated by the flow is therefore proportional to *Q*.

Because the sensing element is located above the inlet/cavity region rather than uniformly along the full channel length, the load acting on the 20 µm PDMS diaphragm is better represented by an effective local pressure:

3 Δ*P*_eff_ = *η*Δ*P*_ch_

where *η* is a geometry-dependent pressure-transfer factor accounting for local pressure redistribution near the inlet and sensing cavity.

The PDMS isolation layer can then be approximated as a clamped elastic diaphragm. In the small-deflection regime, classical plate mechanics indicate that the characteristic strain transferred to the bonded piezoelectric layer increases approximately linearly with the effective local pressure:^34^

4 *ε̄*_m_ = *K*_m_Δ*P*_eff_

where *K*_m_ is a lumped coefficient determined by diaphragm geometry and material properties.

Under small deformation, the open-circuit response of the bonded piezoelectric layer can then be written at the device level as

5 *V*_oc_ ≈ *G*_p_*ε̄*_m_ = *K*_eff_*Q*

where *G*_p_ is an effective electromechanical conversion factor and *K*_eff_ is the overall device-level proportionality constant. Eqn (4) shows that the observed linear *V*–*Q* relation is the first-order coupled consequence of laminar hydraulic resistance, local pressure transfer, diaphragm deformation, strain-mediated stress transfer, and piezoelectric conversion.^32–35^

We further clarify the assumptions and validity range of this first-order model. It is intended for steady, incompressible, low-Reynolds-number laminar flow of dilute Newtonian aqueous electrolytes, fixed device geometry, and small-to-moderate diaphragm deformation with quasi-static membrane response. Within this regime, a linear first-order relation between output voltage and flow rate is expected. In contrast, in highly compliant PDMS microchannels or at larger wall deformation, fluid–structure interaction can produce nonlinear flow–pressure relations and geometry-dependent corrections.^36,37^ Therefore, the above derivation should be regarded as a physically grounded first-order interpretation for the present device architecture and the experimentally tested range of 3-203 µL min^−1^, rather than as a universal predictive law beyond the measured conditions.

To further assess the performance of the proposed device, a detailed comparison with representative recently reported microfluidic flow sensors is provided in Table S2, including detection range, sensitivity, response time, external power requirement, and applicable fluid type. As summarized in Table S2, the main advantages of the present sensor are its self-powered operation, simple device architecture, low-flow detectability (∼3 µL min^−1^), and applicability to conductive electrolyte solutions without requiring direct electrical contact with the fluid. These features are attractive for portable and easily integrated lab-on-a-chip platforms.

At the same time, the present device also has several limitations. Compared with some optical or thermal methods, it does not achieve the lowest detection limit reported in the literature, and the current work focuses on flow monitoring rather than selective identification of different ionic species. In addition, the sensing mechanism is described here using a semi-empirical model within the tested operating range, and a more general predictive model will require further study. Therefore, the main contribution of this work lies in providing a self-powered, structurally simple, and robust solution for low-flow monitoring in conductive microfluidic environments.

### 3.3. Operational stability and environmental robustness

**Fig. 6:**
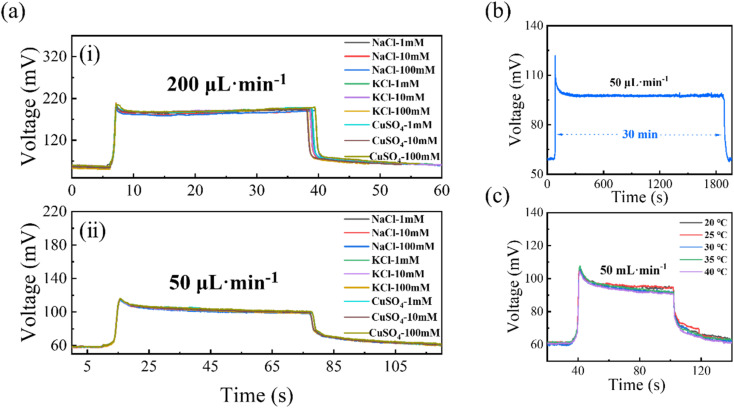
Operational stability and environmental robustness of the sensor. (a) (i) Output voltage responses of the sensor in NaCl, KCl, and CuSO4 solutions with concentrations of 1 mM, 10 mM, and 100 mM at a flow rate of 200 µL min^−1^. (a) (ii) Output voltage responses of the sensor in NaCl, KCl, and CuSO_4_ solutions with concentrations of 1 mM, 10 mM, and 100 mM at a flow rate of 50 µL min^−1^. (b) Voltage output of the sensor during 30 min continuous operation using a 1 mM NaCl solution at a flow rate of 50 µL min^−1^. (c) Temperature-dependent output voltage of the sensor measured over the range of 20–40 °C, showing slight thermal drift under moderate temperature variations.

To further evaluate the operational stability and environmental robustness of the proposed sensor under practical microfluidic conditions, electrolyte-environment tests, long-term continuous-flow measurements, and thermal drift characterization were carried out. As shown in Fig. 6a(i) and (ii), the steady-state output voltages measured in NaCl, KCl, and CuSO_4_ solutions with concentrations of 1, 10, and 100 mM ranged from 79.03 to 81.37 mV at 50 µL min^−1^ and from 165.59 to 173.37 mV at 200 µL min^−1^. Relative to the 1 mM NaCl reference, the maximum deviations were 1.65% and 2.70% (see eqn (2) in the SI for calculation details), respectively, indicating limited electrolyte-dependent variation within the investigated range. These results indicate that the sensing response is mainly governed by flow-induced pressure deformation of the PDMS membrane and the subsequent piezoelectric conversion, rather than by the specific electrolyte composition.

As shown in Fig. 6b, after excluding the startup and shutdown transients, the steady-state output during 30 min continuous operation in 1 mM NaCl at 50 µL min^−1^ was 77.62 ± 0.32 mV, with a drift rate of only −0.0069 mV min^−1^ and a difference of only 0.20% between the first and last 5 min steady-state windows (see eqn (3) in the SI for calculation details), confirming good long-term operational stability.

In addition, the steady-state output voltages were 74.26, 74.92, 71.82, 72.40, and 71.30 mV at 20, 25, 30, 35, and 45 °C, respectively, corresponding to a total variation of 4.0% over 20–45 °C and an average temperature coefficient of −0.136 mV °C^−1^ (see eqn (4) in the SI for calculation details). As shown in Fig. 6c, the sensor also exhibited stable steady-state output under different electrolyte environments, indicating good tolerance to variations in ionic conditions. These quantitative results confirm that the proposed sensor maintains stable sensing performance under prolonged flow, moderate temperature variation, and different electrolyte environments.

## 4. Conclusion

In summary, we have developed a self-powered piezoelectric microfluidic flow sensor for sensitive and stable monitoring of low-velocity metal-ion solution flows. The device utilizes a PDMS isolation diaphragm to mechanically couple the sensing element to the microchannel, enabling fully non-contact flow sensing. Without requiring external power or dynamic excitation, the sensor achieves a low detection limit of ∼3 µL min^−1^ and a broad measurable range of 3–203 µL min^−1^, along with a high sensitivity of 0.79 mV (µL min^−1^)^−1^, a fast response time of 0.1 ms, and excellent durability over 5000 cycles.

In addition, employing a commercial piezoelectric film as the core sensing material offers practical advantages for translation, including reduced development cost, good consistency, and simplified assembly. These advantages enhance the portability and transferability of the sensing concept, facilitating adaptation to different chip geometries and application requirements without relying on bespoke piezoelectric material synthesis or complex microfabrication. The proposed low-power, integrable, and non-invasive device offers a scalable and versatile solution for precise microflow monitoring in lab-on-a-chip systems, biochemical microreactors, and point-of-care diagnostics, thereby laying a robust foundation for next-generation microfluidic sensing technologies.

## Author contributions

Y. F., J. J., and L. C. conceived the ideas. Y. Z., J. J., and Y. F. designed the experiments, and co-wrote the manuscript. Y. Z., T. W., W. L., B. X., S. C., and X. X. performed the experiments and prepared the data graphs. All authors discussed the results and commented on the manuscript.

## Conflicts of interest

The authors declare that they have no competing interests.

## Supplementary Material

RA-016-D6RA02026G-s001

## Data Availability

All data generated or analyzed during this study (including device fabrication details, electromechanical characterization, piezoresponse force microscopy measurements, flow-rate calibration, and microflow sensing results) are provided in the main text. Supplementary information is available. See DOI: https://doi.org/10.1039/d6ra02026g.
