# Supplementary material for: Self-powered piezoelectric microfluidic flow sensor for low-flow monitoring of metal-ion solutions
Source: RSC Adv. 2026 Apr 16;16(22):19842–50. doi: 10.1039/d6ra02026g (PMC13085997; doi:10.1039/d6ra02026g)
Supplement: RA-016-D6RA02026G-s001 [file RA-016-D6RA02026G-s001.pdf]

Supplementary Information (SI) for RSC Advances.
This journal is © The Royal Society of Chemistry 2026

Self-Powered Piezoelectric Microfluidic Flow Sensor for Low-Flow Monitoring of Metal-Ion Solutions

Yunzheng Zhang[1,#], Tao Wang[2,#], Jun Zheng[1,#],Wenjin Luo[3], Zhangjun Lan[4], Binyou Xie[1], Shushu Chen[2], Xinming Xia[3], Liuhua Mu[2], Jie Jiang[2,*], and Yan Fan[1,*], Liang Chen[2]

[1] College of Optical, Mechanical and Electrical Engineering, Zhejiang A&F University, Hangzhou 311300, China.
[2] School of Physical Science and Technology, Ningbo University, Ningbo 315211, China.
[3] College of Physics Science and Technology, Yangzhou University, Jiangsu, 225009, China.

[#]These authors contributed equally to this work.
* Corresponding author. E-mail: fanyan503@zafu.edu.cn, and jiangjie1@nbu.edu.cn

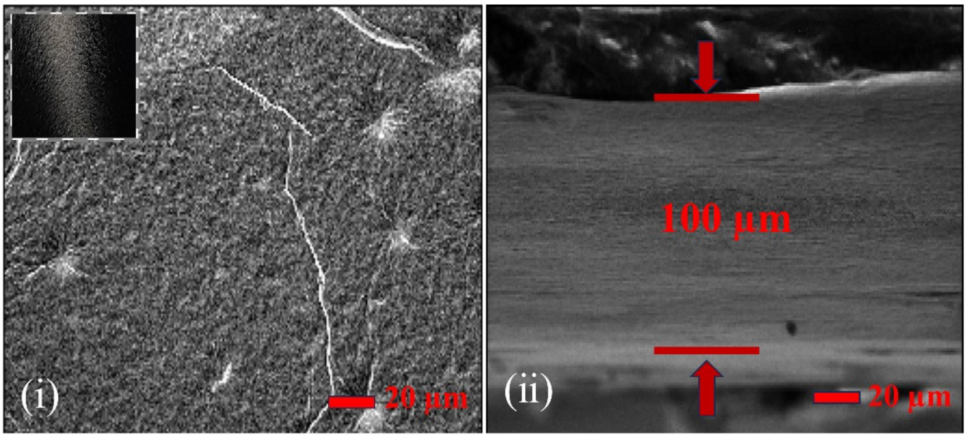

Figure S1. SEM images of the commercial piezoelectric film: (i) plan-view SEM image showing a relatively smooth and uniform surface; (ii) cross-sectional SEM image indicating a film thickness of approximately 100 μm.

Table S1. Gravimetric calibration results of the syringe pump at set flow rates of 3, 10, 50, 100, and 200 μL·min^{-1}.

Set flow rate (μL·min^{-1})	Collection time (min)	Measured flow rate (μL·min^{-1}, mean ± SD, n = 5)	Relative error (%)	RSD (%)
3	60	3.009 ± 0.021	0.30	0.69
10	30	10.032 ± 0.031	0.32	0.31
50	10	50.050 ± 0.274	0.10	0.55
100	10	100.088 ± 0.534	0.09	0.53
200	5	200.124 ± 0.192	0.06	0.19

Note: The actual flow rate was calculated from the collected liquid mass and collection time. Relative error was calculated with respect to the programmed flow rate.

Table S2. Comparison of Representative Microfluidic Flow Sensors

Author	Sensing Method	Sensitivity	Limit of detection	Range	External power	Applicable fluid types
Hongdan Wan et al. (2025)[1]	Optical sensing	~0.27 pm/(μL·min^{-1})	~1.43μL·min^{-1}	-	Yes	-
Adam Hawke et al. (2023)[2]	Pressure sensing	-	-	10–500μL·min^{-1}	Yes	DI water; water-glycerol solutions; blood
Weinan Liu et al. (2025)[3]	Optical sensing	36.5 nm/(μL·s^{-1})	~212nL·s^{-1}	0–1.6μL·s^{-1}	Yes	Water
R. Vilares et al. (2010)[4]	Thermal flow sensing	485μV/(μL·min^{-1})	40nL·min^{-1}	0–25μL·min^{-1}	Yes	DI water
Zijun Zhang et al. (2024)[5]	Pressure sensing	4.33–6.67 Pa/(mL·h^{-1})	2.9μL·h^{-1}	11.8–118.0mL·min^{-1}	Yes	DI water
John Collins and Abraham P. Lee (2004)[6]	Electrical admittance measurement	5.2 × 10^{-4} mA/(mL·min^{-1})	0.05mL·min^{-1}	>0.05mL·min^{-1}	Yes	NaOH; $CaCl_2$; KCl; KOH; D-PBS; D-MEM
Martin Seidl (2024)[7]	Mass flow sensing	-	10μL·min^{-1}	>20mL·min^{-1}	Yes	air
Nadine Noeth et al. (2011)[8]	Optical sensing	-	3nL·min^{-1}	-	Yes	water
Tiange Wu et al. (2020)[9]	Optical sensing	4.65 × 10^{5} mV/(m·s^{-1})	0.7 mm·s^{-1}	0.6–14 kPa	Yes	Blood; DI water
Wenxue Li et al. (2025)[10]	Optical sensing	22.51 nm/(mm·s^{-1})	-	0.17–1.17mm·s^{-1}	Yes	DI water
Alex Baldwin et al. (2016)[11]	Electrochemical sensing	-	-	−400 to 400μL·min^{-1}	Yes	physiological ionic fluids
Harsh Deswal et al. (2024)[12]	Pressure sensing	0.016 Ω/(μL·min^{-1})	5μL·min^{-1}	0–200μL·min^{-1}	Yes	NaCl (0.1–0.6 M）
Trevor Q. Hudson et al. (2021)[13]	Electrochemical sensing	-	-	43–200μL·min^{-1}	Yes	physiological ionic fluids
This work	Pressure sensing	0.79 mV/(μL·min^{-1})	3μL·min^{-1}	3-203μL·min^{-1}	NO (self-powered)	DI water; electrolyte solutions

Section S3. Quantification of stability metrics

To quantitatively evaluate the operational stability of the sensor, the steady-state output voltage for each condition was defined as the arithmetic mean of the selected plateau window[14]:

$$\bar{V}_{ss} = \frac{1}{n}\sum_{i=1}^{n} V_i \qquad (S1)$$

where V_i *is the instantaneous voltage and* n *is the number of data points in the steady-*

state window.

For electrolyte-dependent measurements, the normalized deviation of the steady-state output under the j-th condition relative to the reference condition[14] *was calculated as*

$$\delta(\%) = \frac{\bar{V}_{ss,j} - \bar{V}_{ss,ref}}{\bar{V}_{ss,ref}} \times 100\%, \qquad (S2)$$

where $\bar{V}_{ss,j}$ *is the mean steady-state output voltage under the* j*-th electrolyte condition, and* $\bar{V}_{ss,ref}$ *is the mean steady-state output voltage under the reference condition (1 mM NaCl at the same flow rate). The maximum electrolyte-dependent deviation reported in the main text corresponds to the largest value of* $\delta(\%)$ *among all tested electrolyte conditions.*

For the long-term continuous-flow test, the relative drift magnitude between the first and last 5 min steady-state windows[14,16] *was calculated as*

$$D(\%) = \left| \frac{\bar{V}_{end} - \bar{V}_{start}}{\bar{V}_{start}} \right| \times 100\% \qquad (S3)$$

where $\bar{V}_{start}$ *and* $\bar{V}_{end}$ *are the mean output voltages in the first and last 5 min steady-state windows, respectively, after excluding the startup and shutdown transients.*

the normalized temperature variation relative to the 20 °C condition[15,16] *was calculated as*

$$\eta_T(\%) = \left| \frac{\bar{V}_{ss,T} - \bar{V}_{ss,20\,^\circ C}}{\bar{V}_{ss,20\,^\circ C}} \right| \times 100\% \qquad (S4)$$

where $\bar{V}_{ss,T}$ *is the mean steady-state output voltage at temperature* T*, and* $\bar{V}_{ss,20\,^\circ C}$ *is the corresponding mean steady-state output voltage at the reference temperature of 20 °C. In this work, because the stability datasets correspond to continuous time traces under fixed conditions rather than multiple independent replicate runs, temporal SD/RSD and condition-to-condition deviations were used as quantitative descriptors of signal stability.*

References

1. H. Wan, S. Zhang, X. Chen, Y. Cui, S. Li, Y. Xiao and C. Wan, *Optics & Laser Technology*, 2025, **184**, 112520.
2. A. Hawke, G. Concilia, P. Thurgood, A. Ahnood, S. Baratchi and K. Khoshmanesh, *Sensors and Actuators A: Physical*, 2023, **362**, 114686.
3. W. Liu, S. Pu, C. Zhang, S. Huang, T. Xu and Q. Wu, *Sensors and Actuators A: Physical*, 2025, **393**, 116844.

4. R. Vilares, C. Hunter, I. Ugarte, I. Aranburu, J. Berganzo, J. Elizalde and L. J. Fernandez, *Sensors and Actuators B: Chemical*, 2010, **147**, 411-417.
5. Z. Zhang, T. Hou, Y. Ren, C. Wang, Z. Wang, H. Pan, P. Li, R. Smith and J. Wang, *Flow Measurement and Instrumentation*, 2024, **96**, 102551.
6. J. Collins and A. P. Lee, *Lab Chip*, 2004, **4**, 7-10.
7. M. Seidl and G. Schrag, *Micromachines*, 2024, **15**, 1404.
8. N. Noeth, S. S. Keller and A. Boisen, *Journal of Micromechanics and Microengineering*, 2011, **21**, 015007.
9. T. Wu, J. Shen, Z. Li, T. Zou, W. Xin, F. Xing, F. Zhang, Z. Man and S. Fu, *Opt Express*, 2020, **28**, 16594-16604.
10. W. Li, J. Li, J. Ye, W. Xu, Y. Liu and S. Qu, *Opt Express*, 2025, **33**, 12509-12518.
11. A. Baldwin, L. Yu and E. Meng, *Journal of Microelectromechanical Systems*, 2016, **25**, 1015-1024.
12. H. Deswal, U. Pandey, S. G. Singh and A. Agrawal, *International Journal of Thermofluids*, 2024, **23**, 100760.
13. T. Q. Hudson and E. Meng, *Journal of Microelectromechanical Systems*, 2021, **30**, 456-470.
14. N. Heckert, J. Filliben, C. Croarkin, B. Hembree, W. Guthrie, P. Tobias and J. Prinz, *Journal*, 2002.
15. L. Liu, Y. Dou, J. Wang, Y. Zhao, W. Kong, C. Ma, D. He, H. Wang, H. Zhang, A. Chang and P. Zhao, *Advanced Science*, 2024, **11**, 2405003.
16. J. Li, Z. Fang, D. Wei and Y. Liu, *Advanced Healthcare Materials*, 2024, **13**, 2401532.
